# High throughput viscoelastic particle focusing and separation in spiral microchannels

**DOI:** 10.1038/s41598-021-88047-4

**Published:** 2021-04-19

**Authors:** Tharagan Kumar, Harisha Ramachandraiah, Sharath Narayana Iyengar, Indradumna Banerjee, Gustaf Mårtensson, Aman Russom

**Affiliations:** 1 Division of Nanobiotechnology, Department of Protein Science, Science for Life Laboratory, KTH Royal Institute of Technology, Solna, Sweden; 2 AIMES-Center for the Advancement of Integrated Medical and Engineering Sciences at Karolinska Institutet and KTH Royal Institute of Technology, Stockholm, Sweden

**Keywords:** Microfluidics, Nanobiotechnology

## Abstract

Passive particle manipulation using inertial and elasto-inertial microfluidics have received substantial interest in recent years and have found various applications in high throughput particle sorting and separation. For separation applications, elasto-inertial microfluidics has thus far been applied at substantial lower flow rates as compared to inertial microfluidics. In this work, we explore viscoelastic particle focusing and separation in spiral channels at two orders of magnitude higher Reynolds numbers than previously reported. We show that the balance between dominant inertial lift force, dean drag force and elastic force enables stable 3D particle focusing at dynamically high Reynolds numbers. Using a two-turn spiral, we show that particles, initially pinched towards the inner wall using an elasticity enhancer, PEO (polyethylene oxide), as sheath migrate towards the outer wall strictly based on size and can be effectively separated with high precision. As a proof of principle for high resolution particle separation, 15 µm particles were effectively separated from 10 µm particles. A separation efficiency of 98% for the 10 µm and 97% for the 15 µm particles was achieved. Furthermore, we demonstrate sheath-less, high throughput, separation using a novel integrated two-spiral device and achieved a separation efficiency of 89% for the 10 µm and 99% for the 15 µm particles at a sample flow rate of 1 mL/min—a throughput previously only reported for inertial microfluidics. We anticipate the ability to precisely control particles in 3D at extremely high flow rates will open up several applications, including the development of ultra-high throughput microflow cytometers and high-resolution separation of rare cells for point of care diagnostics.

## Introduction

Microfluidics based particle focusing and separation has been widely utilised in biomedical applications and a number of methods have been developed over the past few decades. These methods can broadly be categorized into active methods, such as dielectrophoresis^1,2^, magnetophoresis^3^ and acoustophoresis^4^, or passive methods, such as deterministic lateral displacement^5^, pinch flow fractionation^6,7^ and inertial microfluidics^8–15^. Among these methods, inertial microfluidics has attracted substantial attention due to extremely high flow rates obtained for particle focusing and separation. Inertial migration of particles across the fluid streamlines has been exploited for high throughput separation applications in rectangular cross-sectioned straight^14^ and curved microfluidic channels^15,16^. Inertial lateral migration of particles in channel flows has been extensively studied in the field of fluid mechanics^17–20^. Segre and Silberberg observed the particles flowing through circular pipes are arranged in the annulus centered at a distance of 0.3 times the diameter of the centimeter-scale pipe cross-section^21^. Particle suspension in Newtonian Poiseuille flow in a straight rectangular microchannel will migrate and the balance between the shear-induced lift forces and wall-induced forces actuates the particles to four equilibrium positions^22^ and by varying the aspect ratio of the channel the equilibrium positions can be changed to two focusing positions^14,23,24^. Owing to the strong correlation between the inertial lift forces and particle size, inertial microfluidics has been used to focus and separate larger particles. Very recently, Toner et al., introduced oscillatory inertial microfluidics to focus smaller particles without the requirement of extremely long channels^25,26^. In a curvilinear channel, the pressure gradient difference in the radial direction will induce a cross sectional secondary flow, consisting of counter-rotating vortices (Dean vortices) above and below the plane of symmetry of the channel, thereby satisfying the mass balance across the inner and outer wall region. The dominating inertial lift forces and the Dean drag force causes particles to migrate and find its equilibrium positions^27–29^. A number of spiral cell-sorting devices have been reported for cell separation according to their size^11,15,16,30–34^. Despite extremely high volumetric flow rates can obtained in inertial microfluidics, separation of smaller particles is challenging. In addition, the fact that particles focus at the centre faces of the walls is not ideal for flow cytometry applications. To address this, particle migration in non-Newtonian fluids has recently been proposed and is gaining substantial interest.

In the case of a pressure-driven, non-Newtonian, viscoelastic flow, particles migrate towards the centreline of a microchannel due to a non-uniform distribution of the first normal stress between the centreline and the walls of microchannel^35^ with non-negligible inertial effects in addition to the dominant elastic forces^36–47^. For 3D focusing of particles in rectangular geometries under similar conditions, the particles migrate towards the centreline and corners of the channel due to nonlinear effects of fluid inertia and fluid elasticity induced by the non-uniform distribution of normal stress. The synergistic combination of fluid elasticity and fluid inertia has been demonstrated for 3D focusing of particles in microchannels with a rectangular cross-section^48^. By using circular cross-section straight channels that exclude corner effects found in rectangular geometries, we previously reported stable single-stream particle focusing in PEO fluids at high Reynolds numbers (Re up to 100)^49^. For curvilinear channels, particle migration in non-Newtonian viscoelastic fluids is more complex and depends on inertial, elastic and Dean drag forces. Lee et al., recently reported a ‘Dean-coupled elasto-inertial focusing band’ in a spiral channel separating 1.5 µm bead and 10 µm bead at low Reynolds number^50^, Nan Xiang et al., studied the particle focusing in low aspect ratio microchannels over a range of flow rates and deduced the defocusing aspects in spiral microchannels^51^, and more recently Yinning et al., showed size-tunable elasto-inertial sorting of five different particles at a flow rate of 160 µl/min^52^. To our knowledge, these works are the only studies done on spiral channels thus far. At present, it is unknown whether viscoelastic flow in curvilinear channels at very higher Reynolds numbers facilitates the particle migration and focusing, which is the focus of this work.

Here, we investigate particle focusing in a viscoelastic fluid in spiral channels at dynamic Reynolds numbers and report focusing and separation at flow rates previously only reported in Newtonian flows. Using PEO (Polyethylene Oxide) as an elasticity enhancer, we systematically analyzed particle focusing in flow through spiral channels by examining the effects of flow rate, channel geometry and viscoelasticity of the solution on the focusing behavior. Two different sized fluorescent particles (10 μm and 15 μm) were used to investigate these effects. Stable particle focusing towards the outer channel walls was observed over a dynamic range of Reynolds numbers. As a proof of principle for particle separation, spiral microchannel with two inlets and two outlets was used to investigate differential migration of the 10 and 15 µm particles.

## Theoretical background

In Inertial focusing (flow through straight channels), migration and equilibrium of particles are mainly due to two forces, shear induced lift forces and wall induced lift force. This shear induced lift force pushes the particles from the center towards the wall and is denoted as F_LS_ which is defined as^53^:

1 $${{\varvec{F}}}_{{\varvec{L}}{\varvec{S}}}\sim \frac{{{{\varvec{U}}}^{2}{\varvec{a}}}^{3}{\varvec{\rho}}}{{{\varvec{D}}}_{{\varvec{h}}}}$$

where U represents volumetric flow rate, a is dimension of the particle, $\uprho$ is the density of the fluid and D_h_ = $(\frac{2 w h}{w+h})$ is the hydraulic diameter of the channel, where w and h are the channel width and height, respectively. When the particles get closer to the wall, wall induced lift force pushes the particles towards the center of the channel. This is denoted as F_w_ which is defined as^53^:

2 $${{\varvec{F}}}_{{\varvec{W}}}\sim \frac{{{{\varvec{U}}}^{2}{\varvec{a}}}^{6}{\varvec{\rho}}}{{{\varvec{D}}}_{{\varvec{h}}}^{\boldsymbol{ }\boldsymbol{ }4}}$$

The resultant lift force has been denoted as F_L_ = F_LS_ − F_W_. For microfluidic channels with curvature, a pressure difference in the channel is formed with high pressure at the outer wall and low pressure at the inner wall. This difference in pressure induces the flow to turn inwards towards the inner wall, thus forming two counter-rotating vortices normal to the bulk flow. These flow structures are called Dean vortices. The drag force caused due to Dean vortices is denoted as F_D_ and defined as^51^:

3 $${{\varvec{F}}}_{{\varvec{D}}}\sim \frac{{4\boldsymbol{ }{\varvec{U}}}^{2}{\varvec{a}}\boldsymbol{ }{\varvec{\rho}}}{{\varvec{R}}\boldsymbol{ }({{\varvec{w}}+{\varvec{h}})}^{2}}$$

The direction and magnitude of F_D_ changes depending on the position of the particle in the vortices.

In addition, in non-Newtonian viscoelastic fluids, the elastic property of the viscoelastic fluid induces an elastic force F_E_ which is denoted as^51^:

4 $${{\varvec{F}}}_{{\varvec{E}}}\sim \boldsymbol{ }8{{\varvec{a}}}^{3}{\varvec{\lambda}}\boldsymbol{ }{(\frac{{\varvec{U}}}{{\varvec{h}}{{\varvec{w}}}^{2}})}^{3}$$

where λ is the relaxation time.

**Figure 1:**
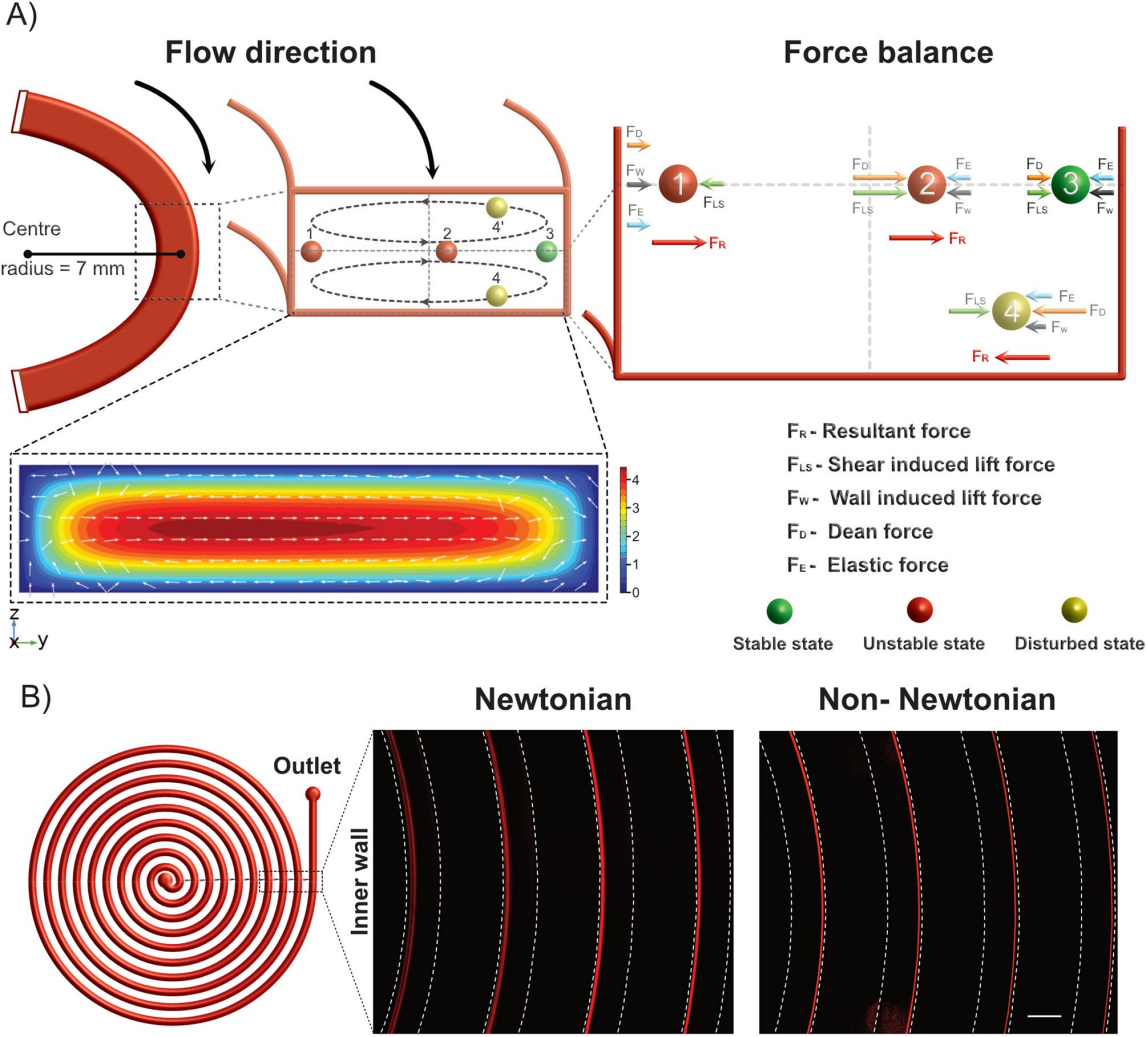
Overview of particle focusing principle in spiral microchannels. (**A**) Schematic illustration of particle focusing in elasto-inertial microfluidics. Under the influence of Dean drag forces (F_D_), particles migrate along the Dean vortices, and depending on the position the particles experience additional strong inertial lift forces (F_LS_ and F_W_) and elastic forces (F_E_). How these forces acting on a particle focused in a distinct point (positions 1–4) are highlighted. Note, there are vertical lift and viscoelastic forces acting on the particles but are negligible at the center line. Inset, COMSOL simulation showing a of a skewed mean flow (contours) and cross-sectional flow (arrows). (**B**) Inertial and elasto-inertial particle focusing. Fluorescence image of 15 µm particles flowing through the spiral in Newtonian (left) and non-Newtonian (right) fluid using PEO as elasticity enhancer. In a Newtonian fluid, the particles are focused at the inner wall and for Non-Newtonian fluid at the outer wall. Scale bar: 500 µm.

Complex interaction and balance between F_L_, F_E_ and F_D_ affect particles and cause them to migrate and find an equilibrium focusing position at the outer wall (Fig. 1A). Qualitatively, to characterize the fluid and particle dynamics during the viscoelastic flow, three dimensionless numbers should be considered: the Reynolds number (Re), Weissenberg number (Wi), and Dean number (De), respectively. The Reynolds number, quantifying the importance of inertia over viscous effects, is defined as Re = $(\frac{\rho U {D}_{h}}{\mu })$, where $\uprho$, U, and μ are the density, average fluid velocity, and dynamic viscosity respectively. The Weissenberg number describes the relative ratio of elastic to viscous properties and is defined as Wi = $(\frac{2 \lambda Q}{h{ w}^{2}})$^50^, where λ is the relaxation time of the polymer additives. The ratio between these two parameters gives the elasticity number El = $(\frac{{W}_{i}}{Re}$), which relates elastic to inertial contributions. Dean number, De = (${Re}_{c }\sqrt{\frac{{D}_{h}}{2R}})$, is a measure of the magnitude of the Dean flow in flows through curved channels. Using COMSOL Multiphysics, we modelled the characteristic flow of curved channel that consisted of a skewed mean flow and the development of a cross-sectional transversally symmetrical cell flow (see Fig. 1 inset). The details of the force components is discussed in more detail in the results.

## Results

### Particle focusing in viscoelastic fluid

In this work, by harnessing the synergetic effects of F_L_, F_E_ and F_D_, we explore elasto-inertial focusing and report stable particle focusing at high flow rates (Re ~ 67). Figure 1A shows schematic illustration of the particles focusing principle. First, let us consider a particle to be at position 1, close to the inner wall. At a sufficiently high flow rate, De increases in magnitude and the particle will start to migrate away from the inner wall. Here, F_D_, F_E_, and F_W_ is counteracted by F_LS_ and the resultant force, which is denoted as F_R_ in Fig. 1A, will push the particles away from the inner wall toward the center. As the particle crosses the center position (at position 2), all other forces change sign except for F_D_. In addition, the magnitude of F_E_ and F_W_ will be decreased and the F_D_ and F_LS_ will increase resulting in pushing the particle away from the center towards the outer wall. At position 3, the particle reaches the lateral equilibrium position and maintain this focused position throughout the remaining channel length. Here, F_D_ is not strong enough to drag the particle along the Dean vortices. However, if the flow rate increases further, De will increase and as a result the particles will be trapped into the vortex and defocus (position 4). Figure 1B shows the difference in particle focusing behavior between Newtonian and non-Newtonian fluid flows. For Newtonian fluid, particles focus close to the inner wall in flow through low aspect ratio spirals, in agreement with previous reports^15^. However, in viscoelastic fluid flow, in addition to the combined effect of lift and Dean forces, elastic forces interplay to focus and order suspended particles at the outer wall^50^.

### The influence of curvature and flow rate

**Figure 2:**
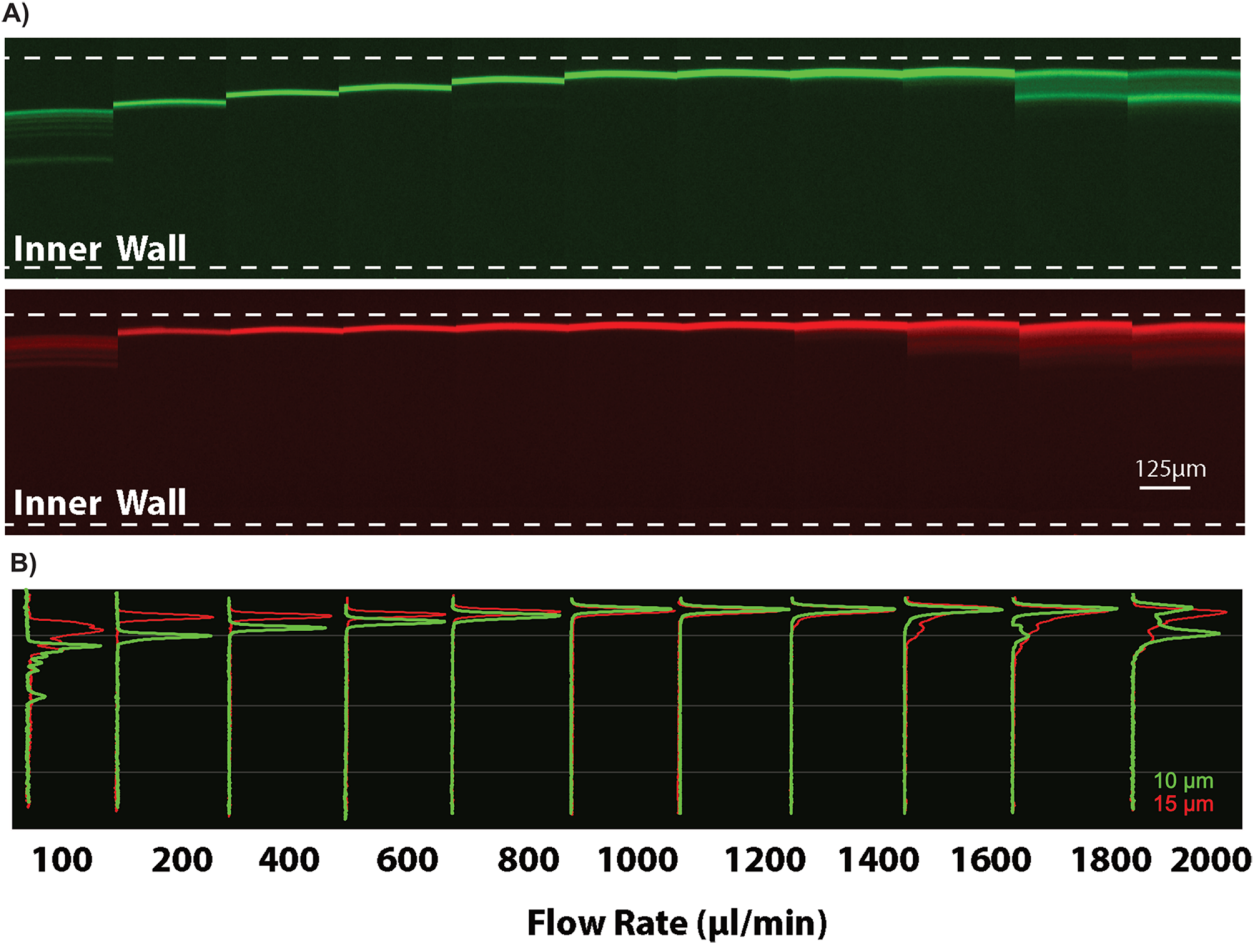
Particle focusing (backward flow direction). (**A**) Fluorescence image of 10 (green) and 15 µm (red) particles at the 5th turn for different flow rates. Stable 3D particle focusing at the outer wall of the spiral microchannel is achieved for a dynamic range of flow rates. Scale bar: 125 µm. (**B**) Corresponding overlapped cross-sectional intensity of the 10 and 15 µm particles. At low flow rates (< 800 μl/min), the larger 15 µm particles are focused closer to the wall and at higher flow rates both the particles are focused at the same lateral position.

To elucidate the particle behavior in viscoelastic fluid flow through curved channels, the effect of different parameters such as spiral microchannel geometries, PEO concentration and flow rate was studied (see S1 in the ESI for the spiral geometries and dimensionless numbers). A ten-turn spiral channel with one inlet and one outlet was used to examine the behavior of 10 µm and 15 µm particles at various flow rates. For convenience, the dimensionless numbers Re, Wi and El for all the channels geometries (for a = 10 µm) at different flow rates are shown in Figure S1B in the ESI. The flow direction is defined from center position (r = 0 mm) as forward and from the outer position (r = 15 mm) as backward flow. Initially, flow from the outer position (backward) was used to evaluate particle focusing at turn 5 (r = 7 mm) for a range of flow rates (Fig. 2). As can be seen in Fig. 2, at low flow rate (100–200 µl/min), although unfocused, the 10 and 15 µm particles start to migrate towards the outer half of the channel width. As the flow rate increases, both particles are clearly focused at the outer wall of the channel. The larger 15 µm particles are initially focused closer to the outer wall compared to the smaller 10 µm particles. However, as the flow rate increases (> 800 µl/min), both particle sizes are fully focused on the same lateral position. As the flow rate increases further the particles start to defocus. The transition is more pronounced for the 10 µm particles, as the particles migrate from a single stream to multiple streams away from the outer wall (at flow rate 1.8–2 ml/min).

**Figure 3:**
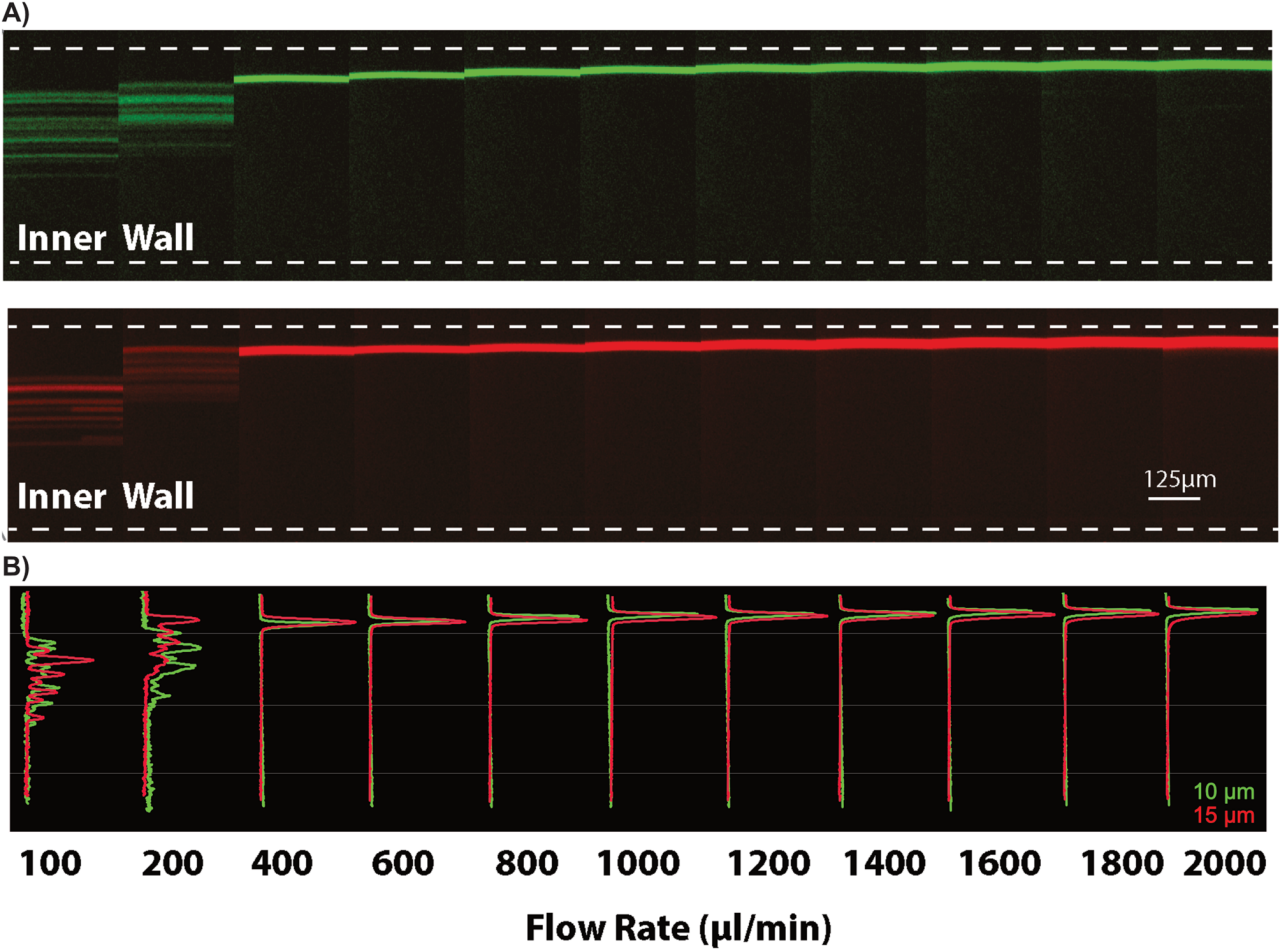
Particle focusing (forward flow direction). (**A**) Fluorescence image of 10 (green) and 15 µm (red) particles at the 10th turn of the spiral for different flow rates. Stable particle focusing obtained at the outer wall of the spiral microchannel for flow rates of 400 µl/min and above. Scale bar: 125 µm. (**B**) Corresponding overlapped cross-sectional intensity of the 10 and 15 µm particles. At low flow rates, the particles are unfocused and spread. As the flow rate is increased, the particles are focused and remain focused even at extremely high flow rates (2 ml/min).

Next, flow in the forward direction was evaluated by observing particle behavior at the outlet (turn 10) (Fig. 3). Initially, the particles were spread and remained unfocused at low flow rate of 100 µl/min (Re < 2), presumably due to insufficient lift and Dean forces. As the flow rate increases both particles are focused at the outer wall. Importantly, the 10 and 15 µm particles are focused at the same lateral position and remain focused even at extremely high flow rate (2 ml/min, corresponding Re = 67 for 10 µm particles).

### The influence of elasticity

**Figure 4:**
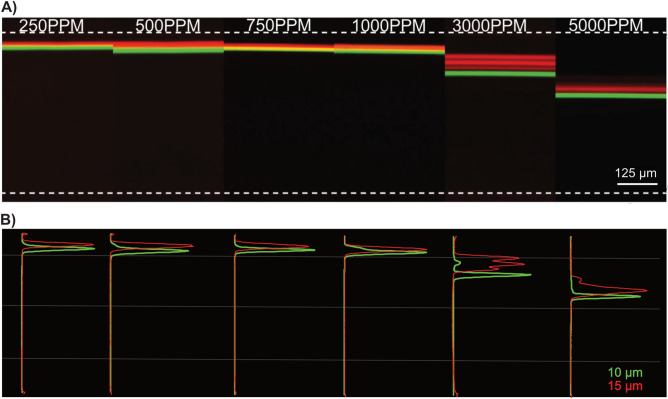
Particle equilibrium focusing position at different PEO concentrations. (**A**) Fluorescence image of 10 (green) and 15 µm (red) particles at the 10th turn for different PEO concentrations. The particle focusing position is pushed away from the outer wall, especially for 3000 PPM and 5000 PPM. Scale bar: 125 µm. (**B**) Corresponding overlapped cross-sectional intensity of the 10 and 15 µm particles.

To investigate the effects of viscoelastic contribution on particle focusing, different concentrations of the PEO solution were tested. The flow rate was kept constant at 300 µl/min, while the PEO concentration was varied from 250 to 5000 ppm. As can be seen in Fig. 4, with increased PEO concentration, the particle focusing position is pushed further away from the outer wall. Furthermore, the 10 µm particles are fully focused, although further away from the outer wall, while the 15 µm particles are more spread at PEO concentrations of 3000 PPM and 5000 PPM. As the PEO concentration increases, the influence of F_E_ on particle is increased and counteracting the influence of F_D_. Consequently, the particles initially fully focused close to the outer wall at lower concentrations experiences larger F_E_ at higher concentrations and are pulled toward the center.

### The influence of blockage ratio on particle focusing

**Figure 5:**
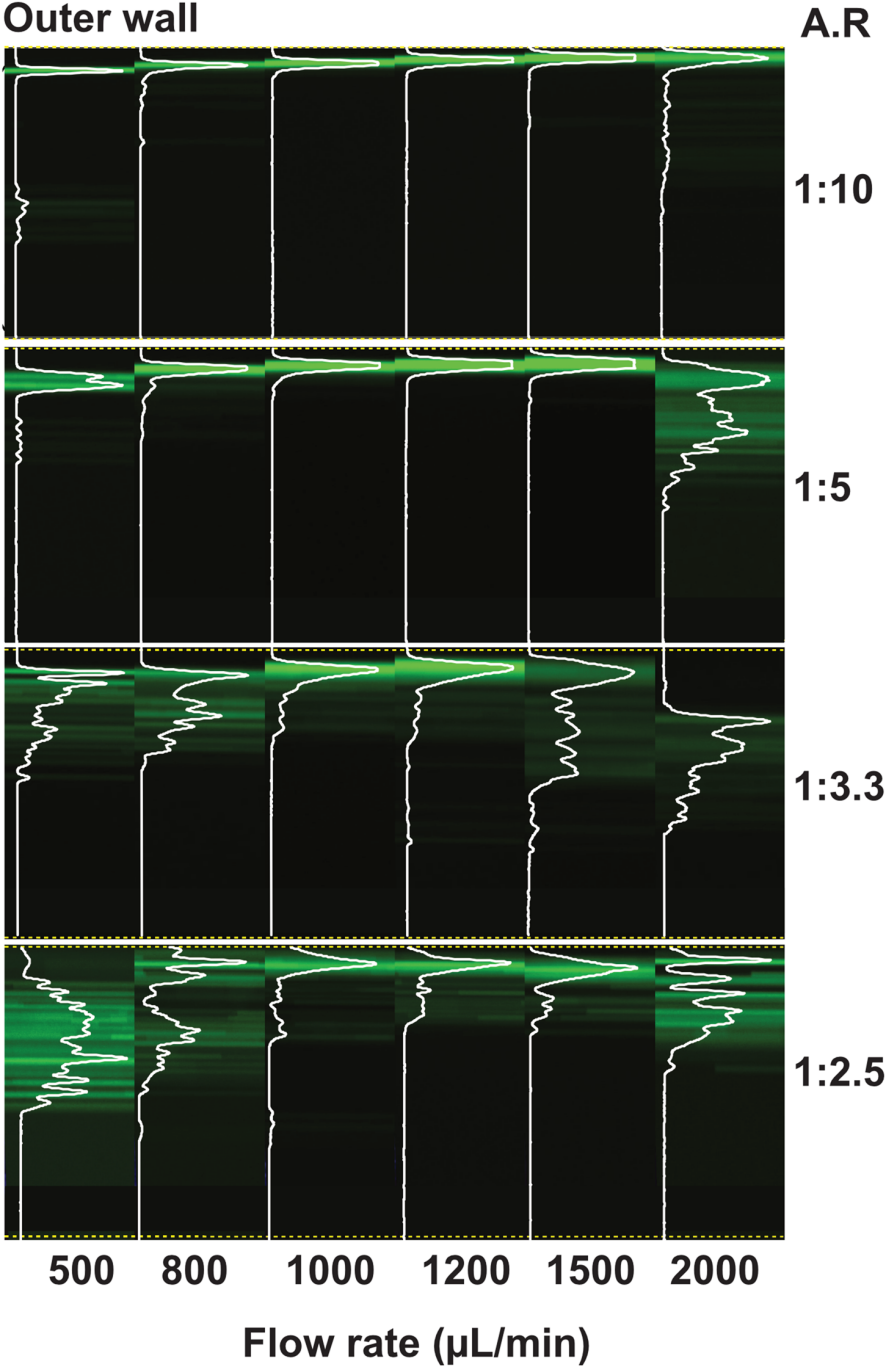
Particle focusing as a function of channel aspect ratio. Fluorescence images of 10 µm particles are overlapped with corresponding cross-section intensity to illustrate the focusing behavior at different flow rates. The lower the aspect ratio the better particle focusing is observed. Flow through low aspect ratio (AR: 1:10) result in stable 3D single stream focusing across the different flow rates tested. For the higher the aspect ratio (AR: 1:5–1:2.5), three regimes can be distinguished: at low flow rate, the particles are spread and at 1 ml/min particles are focused at the outer wall and further increase in flow rate results in defocusing of the particles.

Next, the effect of the channel aspect ratio (AR) i.e., the ratio of the height to the width of the channel (h/w) on particle focusing was studied. The width of a two-turn spiral (see S1-A in the ESI for the spiral geometry) was kept constant at 500 µm, while the height was varied to obtain different AR (1:2.5–1:10). For AR 1:10, particle focusing is observed over a wide range of flow rates (up to 2000 µl/min, with corresponding Re up to 67 (Fig. 5). Increasing the AR results in particle defocusing, especially for AR 1:3.3 and 1:2.5. For AR 1:5 defocusing is observed at the flow rate of 2000 µl/min with a corresponding Re of 61. The difference in particle focusing is more pronounced for the lowest (500 µl/min) and highest (2 ml/min) flow rate tested. At low flow rate (corresponding Re: 26, 24, 22 and 21), a lower AR will result in increased focusing at outer wall while for AR 1:3.3–1:2.5 no focusing is observed. At the highest flow rate (corresponding Re: 67, 61, 56 and 52) particle defocusing is observed in all AR except for 1:10.

**Figure 6:**
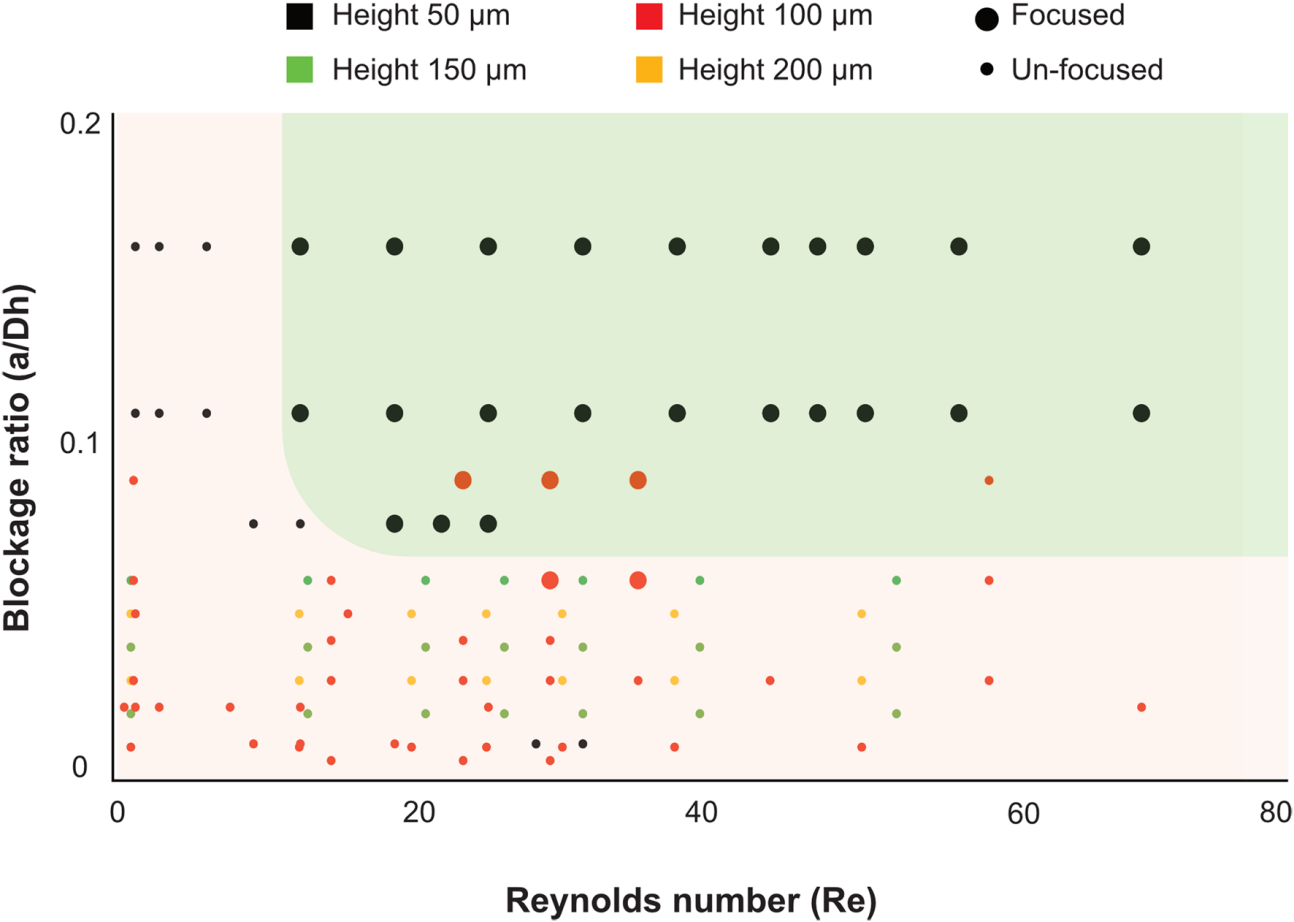
Particle focusing as function of blockage ratio and Re. Graph of particle size to channel size (a/Dh) ratio plotted against Re. Particle focusing is observed at Re greater than10 and a blockage ratio greater than 0.06.

Investigating the particle blockage ratio, defined as the particle size relative to the channel size (a/Dh) is a good measure of the effect of channel geometry and particle size on focusing. In Fig. 6, the blockage ratio values are plotted against Re for a range of particle sizes and channel geometries. The PEO concentration was kept constant at 500 PPM while the channel geometry and flow rate were varied. At low Re (i.e. insufficient lift and Dean forces), all particles remained unfocused. At intermediate Re, focusing is observed as a result of balance between the acting forces (F_L_, F_D_ and F_E_). Our results suggest a minimum blockage ratio > 0.06 for focusing. At high Re (> 60), a blockage ratio > 0.1 is required for particle focusing.

### High throughput and high-resolution particle separation

**Figure 7:**
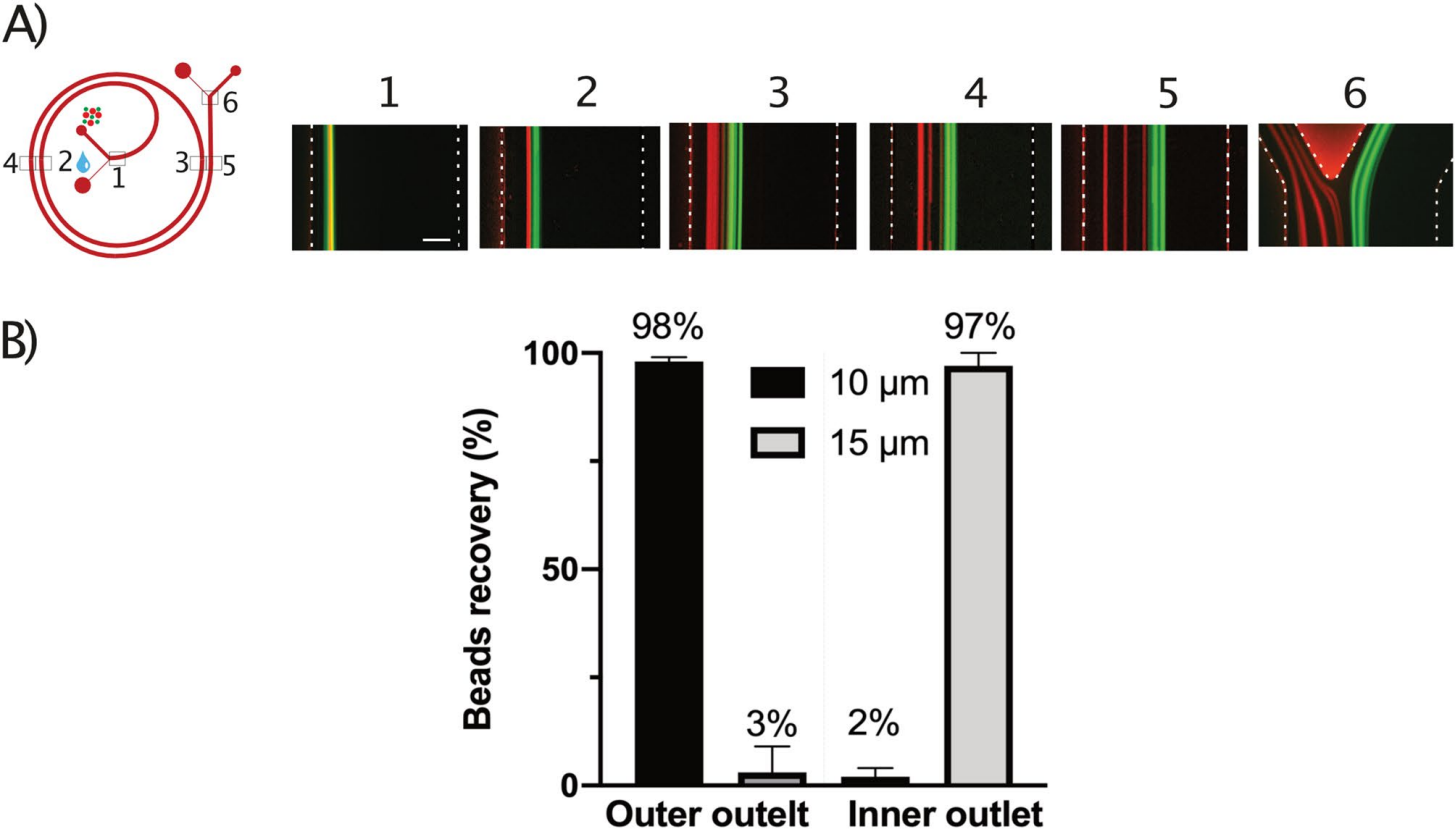
Sheath-based flow for particle separation. (**A**) Differential migration of 10 µm (green) 15 µm particles (red) from the inner wall. Particle position at the different regions (position 1–6) are shown, clearly indicating that the smaller 10 µm particles migrate first towards the outer wall. (**B**) Particle counting results, indicating high separation efficiency where 98% of 10 µm particles were collected at outer outlet and 97% of 15 µm particles at the inner outlet. The total flow rate was 1 mL/min (sheath: 950 µL/min and sample: 50 µL/min).

In order to determine the working conditions for particle separation, a two-turn spiral with two inlets and two outlets was used (see S1-A in the ESI). The PEO concentration was kept constant at 500 PPM for both sample and sheath and different flow rates were tested. Using a sheath flow, the particles are initially pushed towards the inner wall. The three acting forces (F_L_, F_D_ and F_E_) scale differently with particle size. Consequently, the larger particles will experience higher shear induced lift force F_LS_, while the smaller particles will start to migrate towards the outer wall. In Figure S3 in the ESI, differential migration of three different particles sizes (5 µm, 10 µm and 15 µm) is shown. The 5 µm followed by the 10 µm particles are migrating faster while the larger 15 µm particles are lagging. Figure 7A shows the behavior of 10 (green) and 15 µm (red) particles in the presence of a sheath fluid. Initially, all particles are forced to the inner wall by the sheath. The 10 µm particles migrate away from the inner wall first, while the larger 15 µm particles lag and can be separated through the two outlets. 98% of the 10 µm particles are collected from the outer outlet fraction, while 97% of the 15 µm particles are collected from the inner outlet (Fig. 7B). To fully exploit the scaling factor and differentially migrate the particles based on size, as shown in S4 in the ESI, a sheath flow is necessary to initially position the particles into a narrow stream at the inner wall. Note, although it is possible to focus the particles using the same geometry and similar flow conditions, it was not possible to fully separate the 10 and 15 µm particles using inertial microfluidics (Newtonian flow), see S5 in the ESI. All in all, using a sheath flow by carefully optimizing the geometry, PEO concentration and flow rate it is possible to achieve high resolution particle separation in elasto-inertial microfluidics.

**Figure 8:**
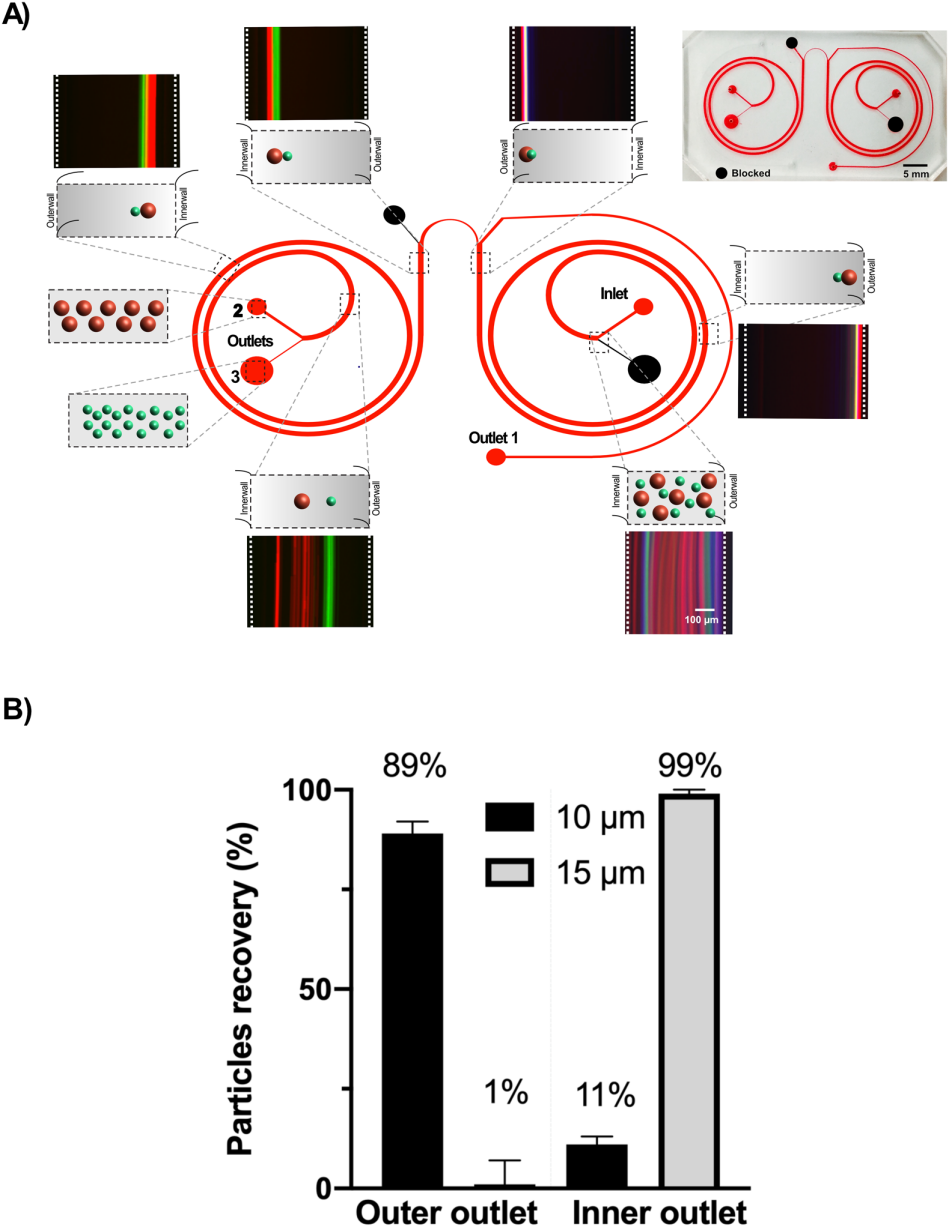
Sheath-less flow for high throughput particle separation using integrated spiral. Experimental results of two integrated spiral. (**A**) Particle size with 10 and 15 μm introduced at the inlet, where 10 and 15 μm particles are pre-focused in the first spiral and follows to the next spiral, where the 15 μm particles remains at the inner wall, while the 10 μm particles moves towards the outer wall of the channel. (**B**) Separation of particle 10 and 15 μm at a high flow rate of 1 ml/min, indicating a separation efficiency of 89% for 10 µm particles at the outer outlet and 99% for 15 µm particles at the inner outlet.

While sufficiently high separation efficiency (98% and 97% for 10 µm and 15 µm particles) was achieved, the overall sample throughput was only 50 µL/min. As described above, this is because there is a need to use a sheath flow to pinch the inlet to position the particles at the inner wall for differential migration, which consequently dramatically decreases the total throughput. To improve the throughput, we developed a novel integrated two-spiral concept (Fig. 8). In the first spiral, both 10 and 15 μm particles are focused towards the outer wall and continue to the second spiral (Fig. 8A). By designing a sharp “U-turn” connecting the two spirals, the particles migrate from outer wall of spiral one towards the inner wall of the second spiral. To evaluate the separation efficiency of the system, a mixture of 10 and 15 μm particles was pushed through a 100 μm height dual-spiral integrated device at a flow rate 1 mL/min. Figure 8A illustrates the different steps involved in the particle separation. Here, the smaller 10 μm particles (green) drift further away from the inner wall after reaching the second spiral compared to the 15 μm particles (red) and can be collected in separate outlet. Figure 8B shows the separation efficiency, as analyzed by coulter counter. 99% of the 15 μm particles were recovered through the inner outlet (outlet2), while 89% 10 μm particles, could be recovered through the outer outlet (outlet 3).

## Discussions

Inertial and elasto-inertial particle focusing has attracted substantial attention in the microfluidic community since the early works of Di Carlo et al.^8^ and Leshansky et al.^35^, who implemented particle inertial and viscoelastic migration, respectively, in microchannels for particle separation. While a growing number of studies have been devoted to flow through straight channels in the case of viscoelastic fluid, there has been very little attention to flow through spiral channels. Also, different with previous works, here for the first time, we describe a high throughput elasto-inertial focusing at Re previously only described in Newtonian flows to precisely control and sort particles. In inertial microfluidics, the interplay between two dominant forces, F_D_ and F_L_, is exploited to focus and order particles. Depending on the relative magnitude of the F_D_ and F_L_ acting on a particle, focusing (dominant lift) or mixing (dominant Dean flow) can occur. In viscoelastic flows, an additional viscoelastic force, F_E_, will interplay with the particles shifting the equilibrium focusing position to the outer wall. Figure 1A shows a schematic illustration of how the three dominant forces, F_L_(F_LS_ and F_W_), F_E_ and F_D_, can interplay to trap particles flowing through a curved channel at an equilibrium position at vertically center point at the outer wall. While the schematic Fig. 1 is simplified, the interplay between these forces is complex and the magnitude of these forces across the channel varies and is currently not fully known. Furthermore, the visco-elastic nature of the fluid leads to decreased magnitude of F_D_ compared to a Newtonian fluid for the same Re. This will also affect the shear induced lift since the parabolic velocity flow profile is flatten at the center, leading the decreased shear at the center. In flow through spiral channel, the radius of curvature affects the stability of particle focusing. In other words, by changing the De (De ∝ 1/R, where R is the radius of curvature), it is possible to affect the relative magnitude of F_D_ with constant Re. For instance, in the backward flow direction, De is increased along the flow and will result in dominance of F_D_ over F_L_ and F_E_ at lower Re compared to forward flow direction. As can be seen in Figs. 2 and 3, in the forward flow direction the particles are fully focused at a flow rate of 2 ml/min, while in the reversed flow direction the particles start to be disturbed. In Figure S2 in the ESI, particle focusing at different turns of the spiral is shown for both flow directions. In addition to the radius of curvature, the ratio of the particle size to the channel size (blockage ratio) plays a key role in the focusing behavior (Fig. 6). Experimentally, we found that a blockage ratio > 0.06 result in particle focusing. Our observation is in agreement with previous work in inertial focusing, where Di Carlo et al. reported blockage ratio > 0.07 for particle focusing^8^. At lower blockage ratios, the particles are too small to be affected by the lift forces. In this work, we demonstrate stable particle focusing at a large dynamic range of flow rates (Re: 20–67).

Continuous flow separation techniques are attractive due to their ability to achieve high throughputs^10,15,16,30–33^. Spiral microchannel based inertial microfluidics is ideally suited for high throughput applications due to the fact that inertial and dean drag forces acting on particles increase with increasing flow rates. However, using a viscoelastic fluid it is possible to fine tune particle focusing using the viscoelastic property of the fluid to achieve stable 3D focusing over a large dynamic range of flow rates^48,50,54^. The lateral particle focusing position is largely affected by the viscoelastic property of the polymer solution (Fig. 4). Experimentally, to evaluate the effect of PEO concentration 300 µl/min was used because of the relatively high pressure drop in the device for high PEO concentrations. A shift in lateral focusing position away from the outer wall is observed for increased PEO concentration. The lateral focusing position was relatively similar for the PEO concentrations between 250 and 1000 ppm. For the higher concentrations (3000 and 5000 ppm), the particle focusing position is significantly pushed towards the center. At the relatively low flow rate used, F_E_ dominates over F_D_ and F_L_. As discussed above, F_L_, F_D_ and F_E_ interact with particles differently depending on the particle position. For high resolution particle separation, the fact that the forces scale differently with respect to particle size (F_L_ and F_E_ ∝ a^3^ versus F_D_ ∝ a) and flow rate can be exploited to differentially migrate particles based on size. Using a sheath flow, we first pre-position the particles close to the inner wall. The increased dominance of F_L_ (F_L_ ∝ a^3^) for the larger particles will enable differential migration of the smaller particles away from the inner wall by forcing the larger particles to be lagging (see S3 in the ESI). Eventually all particles above a certain size cut-off will find the equilibrium focusing position at the outer wall. By exploiting the fact that larger particles are lagging in the migration, particle separation could be successfully demonstrated. High separation efficiency (∼ 98%) was achieved between 10 and 15 µm particles (Fig. 7).

Spiral devices have extensively been studied for particle separation in Newtonian flows. In non-Newtonian flows however, to our knowledge, a recent study has been reported where, Yinning Zhou et al., separated 3, 5 and 10 μm particles at low flow rate of 160 µL/min^52^. In this work, we explore elasto-inertial focusing not only to focus and separate particles with large size differences but to also to differentially focus and separate pre-focused particles. As showed in Fig. 3, the 10 and 15 μm particles are closely focused at lateral position towards the outer wall, which is difficult to separate from each other. Here, we utilize the balance between the three main forces involved to keep the larger 15 μm particles close to the inner wall using a sheath flow while migrating the smaller 10 μm particles towards the outer wall. This way, we can dictate a unique condition where the 15 μm particles are “kept” at the inner wall due to lift dominating over Dean forces (F_L_ > F_D_). While the total flow rate for separation of 10 µm and 15 µm particles was impressive 1 mL/min (Fig. 7), the actual flow rate of the sample was 50 µl/min. For separation applications, the actual sample flow rate is important and has to be accounted for. On the other hand, as shown in S3 and S4 in ESI, sheath flow is important for “pre-positioning” the particles at the inner wall for differential migration and separation. To this end, we designed an integrated two spiral device that enables pre-focusing of the particles in the first spiral and then differentially migrate the smaller particles towards the outer wall while the larger particles are lagging (Fig. 8). Due to the reduction of the flow rate, the relative magnitude of F_D_ is counter balanced by the shear- induced F_L_ directed towards the inner wall. The larger particles are affected more by F_L_ (F_L_ ∝ a^4^ while F_D_ ∝ a). Since the flow is inward, F_D_ start to successively become dominant and then drag the particles towards the outer wall with the smaller particles migrating before the larger particles and can be separated. The novel dual-spiral design not only enable pre-focusing but also volume reduction (almost 50% volume reduction at outlet 1). Using the current design, 10 mL of sample would take 10 min to process and the collected sample volume would be reduced to 2.35 mL for the 15 µm particles and 2.80 mL for the 10 µm particles. The dual spiral device was designed to couple two identical single spirals through a sharp u-shaped channel, such that the first spiral accomplishes complete focusing of both the larger and smaller particles into one focusing position while the second spiral differentially migrate the smaller particles away from the inner wall while the larger particles will be lagging behind for effective separation. In the u-shaped section, the pre-focused particles are entrapped into the strong dean vortices and forced to migrate towards the inner wall of the second spiral. Note, the width of the channel at the u-shaped region is smaller which enables remained focusing despite almost 50% volume reduction via outlet 1. While the novel dual-spiral system is generic design and nicely illustrates proof of principle for pre-focusing, volume reduction and differential particle separation, each of the spiral section can be further optimized for specific applications. For instance, the footprint of the first spiral can be significantly shortened and only have one inlet channel followed by a second spiral without the sharp u-shaped section, but instead the second spiral would twist towards the other direction with two outlets. While outside the focus of this work, we are currently exploring these designs for applications in sepsis diagnostics. Although only separation of two particles sizes has been demonstrated in the current work, we envision separation larger number of particle sizes simultaneously should be possible by further optimizing the channel geometry and extending the outlets. Hence, we expect the focusing dynamics presented in this work will be advantageous for future applications requiring high resolution and high throughput sample processing. We envision the integrated two spiral system to be particular useful for the isolation of circulating tumor cells from peripheral blood for applications in cancer diagnostics.

## Conclusions

In this work, we report a spiral microchannel-based viscoelastic platform for continuous particle focusing and separation at high throughput. Here, we report elasto-Inertial microfluidics working at the flow rate previously only been reported in Inertial microfluidics, with the added benefit of 3D focusing at a single position vertically^48,50,54^. Experimentally, we evaluated particle focusing behaviors over a large dynamic range of flow rates, channel geometry and particle sizes. In addition to stable 3D focusing at two orders magnitude higher Re than previously reported in flow through spiral channels, by carefully adjusting the forces acting on particles it is possible to differentially migrate and separate particles at high resolution. As a proof of principle, we demonstrate differential migration and separation of 10 µm from 15 µm particles at a total flow rate of 1 mL/min. A separation efficiency of 89% for the 10 µm and 99% for the 15 µm particles was achieved. The ability to precisely control particles in 3D at extremely high flow rates will not only open possible applications in high throughput cell separation, but also in the development of low-cost, microflow cytometers.

## Materials and methods

### Device fabrication

For the fabrication of PDMS devices, the designs of the microchannels were created using AutoCAD software and printed on a Mylar mask and standard lithography techniques were used to fabricate the master mold. Briefly, SU-8 a negative photoresist, was spun onto a silicon wafer and exposed to UV light through the mylar mask and developed in microresist SU-8 developer to produce a spiral channel master mold. To generate the PDMS replica, a ratio of 10:1 sylgard 184 elastomer with curing agent was poured over the SU-8 master, after thermal curing for 6 h at 65 °C, the PDMS slabs were cut and punched to produce the inlet and outlets. The cut PDMS slabs were bonded onto the glass surface after brief exposure to oxygen plasma.

### Sample preparation and flow experiments

For Newtonian experiments the particles were suspended in 1× PBS with small amount of tween-20 surfactant. PEO (Poly (ethylene oxide)) was used as an elasticity enhancer for the Non-Newtonian fluid. PEO (Poly (ethylene oxide)), (Mw = 2 000 000, Sigma-Aldrich) was added to deionized water to prepare the following concentrations: 250 ppm, 500 ppm, 750 ppm, 1000 ppm, 3000 ppm and 5000 ppm. For majority of the experiments a concentration of 500 ppm was used for both sample and sheath flow. The PEO solution is considered to have a constant shear viscosity of 1.8 mPa S under the present experimental conditions and its relaxation time is 0.7–1.2 ms^55^.

Spherical, polystyrene fluorescent beads (ThermoFischer Scientific) with diameters 5 µm (green), 10 µm (green) and 15 µm (red), were used in the experiments. Particle suspensions were prepared by spiking particles into the viscoelastic fluid. The flow experiments were performed using a mid-pressure (neMESYS CETONI GmbH) syringe pump. A Coulter counter (Beckman coulter- Z2 coulter particle count and size analyzer) was used for quantification of particles collected from the different outlets after separation and fluorescent imaging was accomplished using an automated Nikon Inverted microscope with Zyla sCMOS Camera. ImageJ, NIH software was used to create and analyze the fluorescent images.

## Supplementary Information

Supplementary Information.
