# Supplementary material for: High throughput viscoelastic particle focusing and separation in spiral microchannels
Source: Sci Rep. 2021 Apr 19;11:8467. doi: 10.1038/s41598-021-88047-4 (PMC8055915; doi:10.1038/s41598-021-88047-4)
Supplement: Supplementary file 1 — Supplementary Information. [file 41598_2021_88047_MOESM1_ESM.docx]

Supplementary Material (ESI) for Scientific reports

Electronic Supplementary Information (ESI)

High throughput viscoelastic particle focusing and separation in spiral microchannels

Tharagan Kumar^a+^; Harisha Ramachandraiah^a+^; Sharath Narayana Iyengar^a^; Indradumna Banerjee ^a^; Gustaf Mårtensson ^a^; Aman Russom ^a,b*^

^a^ KTH Royal Institute of Technology, Division of Nanobiotechnology, Department of Protein Science, Science for Life Laboratory, Solna, Sweden.

^b^ AIMES - Center for the Advancement of Integrated Medical and Engineering Sciences at Karolinska Institutet and KTH Royal Institute of Technology, Stockholm, Sweden

^+^ Shared authors

^*^ Contact author: aman@kth.se (A. Russom)

**Table of Contents**

1. **Spiral designs and dimensionless numbers**
2. **Particle behavior at increasing and decreasing curvature of the spiral**
3. **Differential migration of particles**
4. **Effect of no sheath fluid**
5. **Spiral designs and dimensionless numbers**

Three spiral designs used to study the behavior of particles has been shown in Figure S1-A. A 10-turn spiral channel and two, 2-turn spiral channels, one with single inlet and outlet and the other with two inlets and outlets were used for the experiments. The corresponding dimensions, such as radius, width and height for each design is highlighted. Table in Figure S1-B summarizes the calculated values of dimensionless numbers such as Re, Wi an El at different flow rates for a two-turn spiral channel design having different aspect ratio.

***
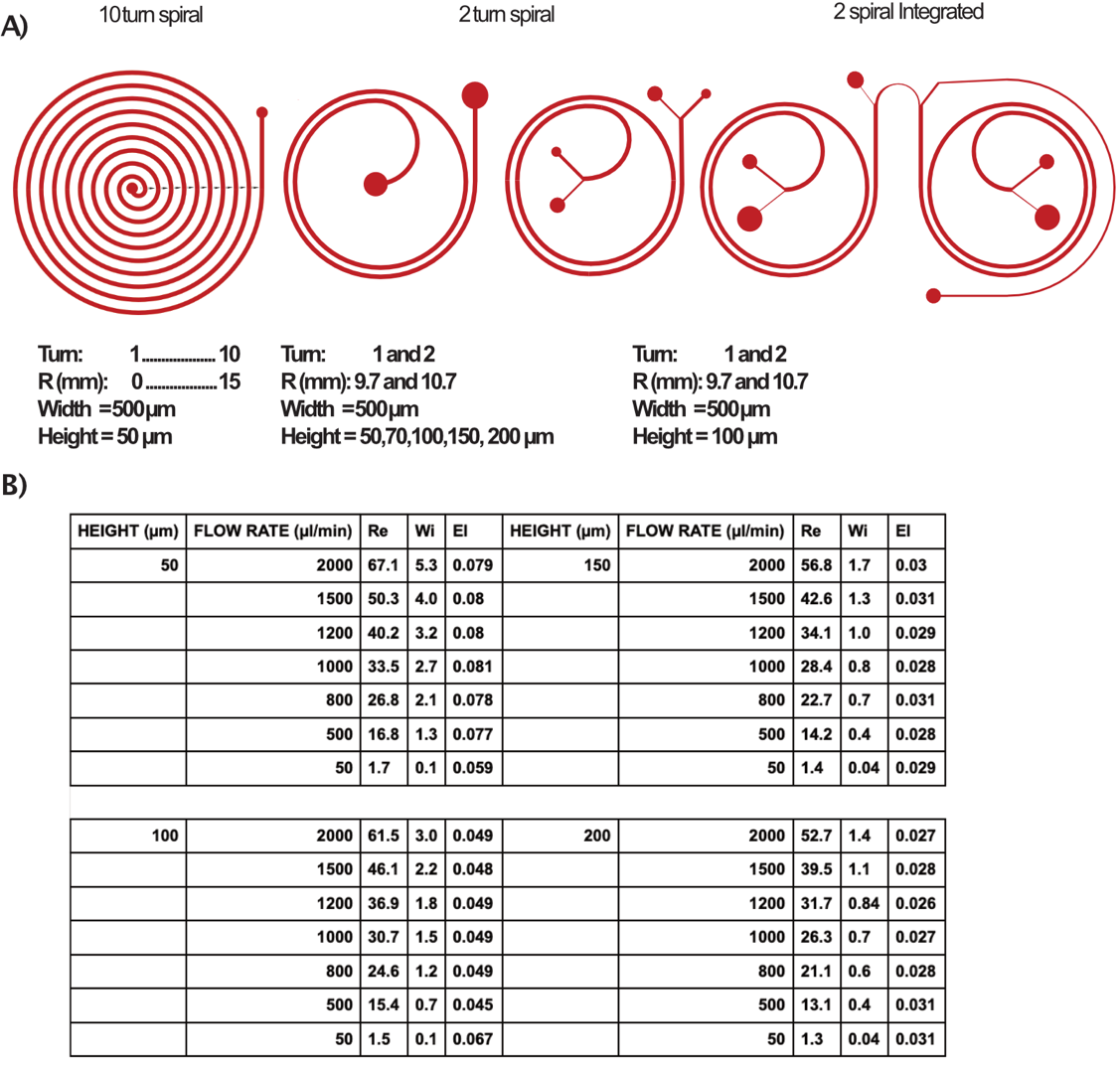
***

**Figure S1.** (A) Channel dimensions, radius, width, and the height of the three spiral channel designs used in the experiments are shown. (B) Table showing values of dimensionless numbers. Re, Wi and El for the heights 50, 100, 150 and 200 µm for different flow rates for a 2-turn spiral design are shown.

1. **Particle behavior at increasing and decreasing curvature of the spiral**

The behavior of 10 (green) and 15 µm (red) particles at each turn for both forward and backward flow for three different flow rates in a 10-turn spiral channel is shown in Figure S2. For forward flow, it can be observed that, at higher flow rates (1 and 2 mL/min), both 10 and 15 µm particles spread at the 2^nd^ turn, and as they reach the 10^th^ turn the particles are fully focus at the outer wall. In contrast, using the same spiral channel but by reversing flow direction, due to the decreasing radius of curvature in backward flow, particles start to spread as observed at flow rates 2000 µL/min due to dominant F_D_.


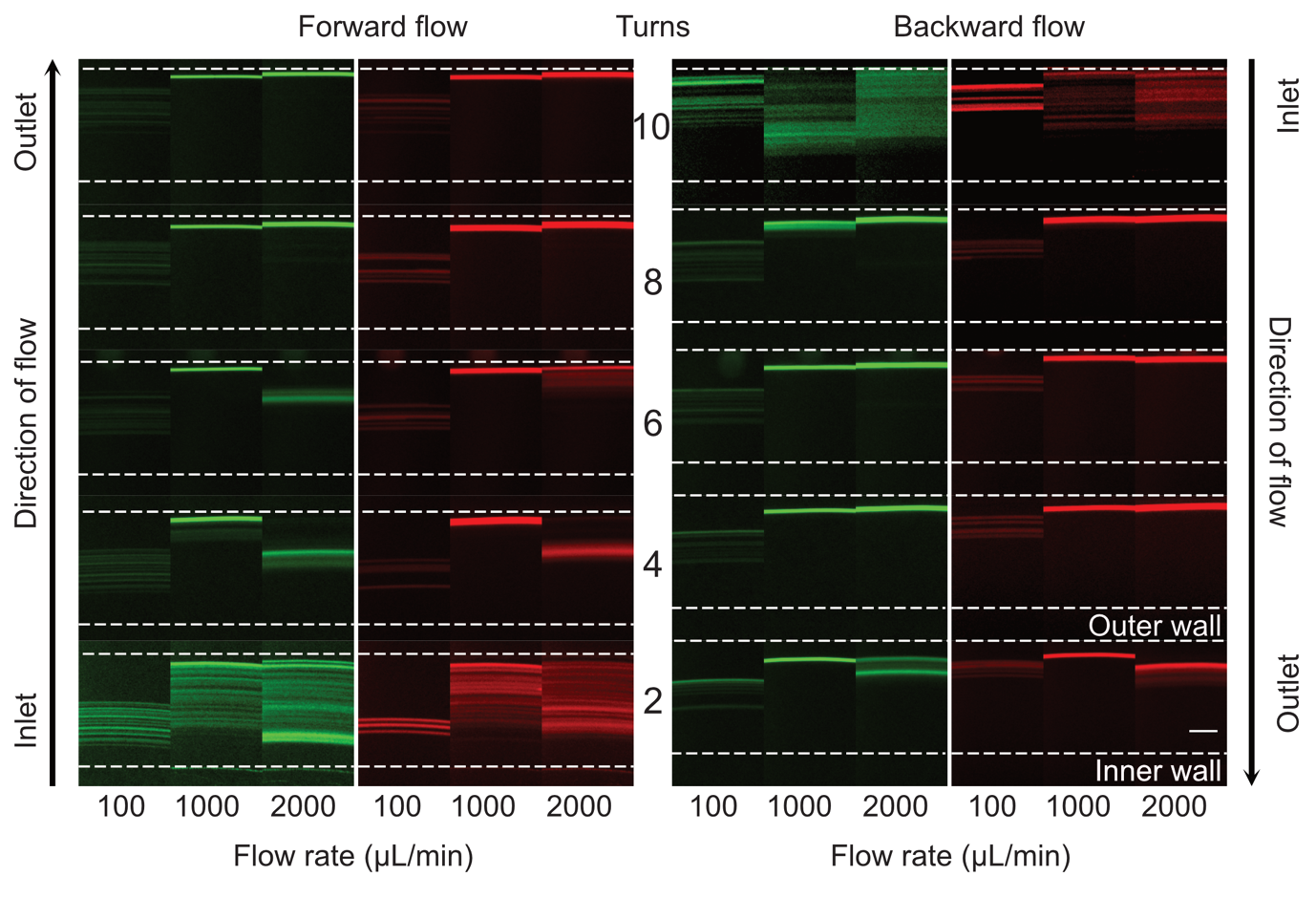


**Figure S2.** The behavior of 10 (green) and 15 µm (red) particles at different turns for forward and backward flow scenarios. At higher flow rates both 10 and 15 µm particles are well focused at the outer wall for forward flow scenario at the 10^th^ turn. However, in backward flow scenario, a decrease in radius of curvature would increase the influence of Dean flow at higher flow rates leading to a spread of the particles.

1. **Differential migration of particles**

The behavior of 5 (blue), 10 (green) and 15 µm (red) particles using a sheath flow is shown in Figure S4. The higher flow rate of the sheath, positions the particles at the inner wall of the inlet. It can be observed that larger particles stay closer to the inner wall while smaller particles will migrate further toward the outer wall of the channel. This is because of dominance of F_L_ on larger particles keeping them close to the inner wall while the smaller particles get entangled in the Dean vortices thus migrating toward the outer wall.


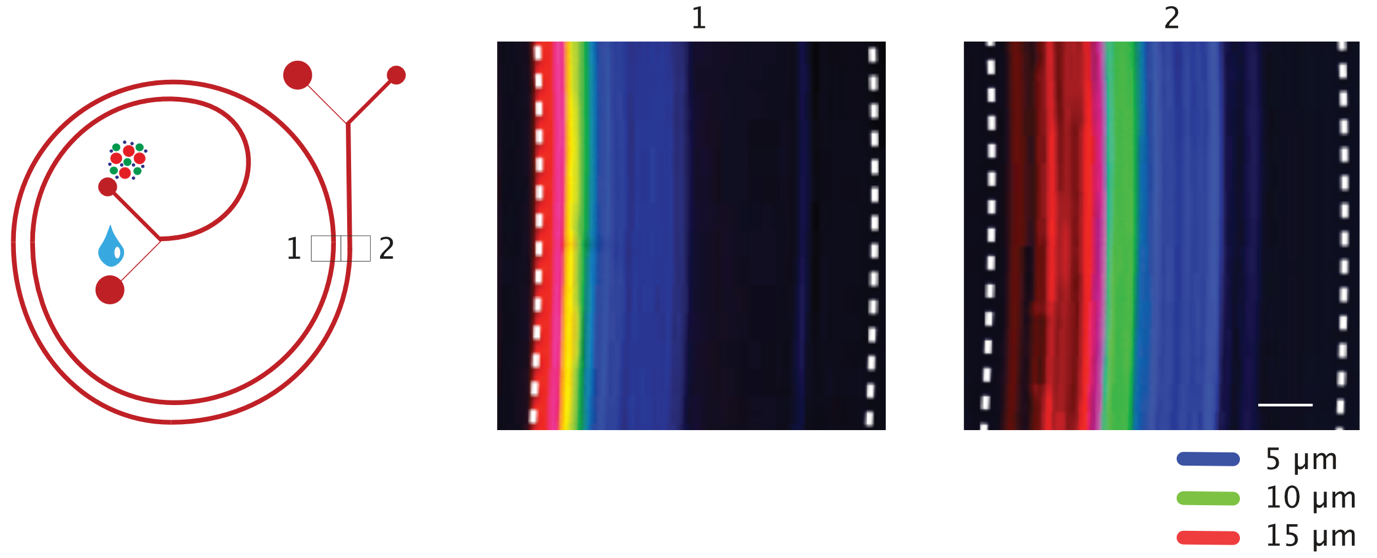


**Figure S3**. Lateral positions of 5 (blue), 10 (green) and 15 µm (red) particles in two turn spiral. Using a sheath flow, the particles are pre-positioned close to the inner wall. The migration pattern can be clearly seen, as bigger the particle stays closer to the inner wall.

1. **Effect of no sheath fluid**

The behavior of 10 (green) and 15 µm (red) particles in a two turn spiral channel is shown in Figure S4. The sample containing particles was introduced at the inner wall of the inlet. In a sheath-less case, particles are distributed uniformly at the inlet of the channel as shown at position 1 in the figure S4-A. At a high flow rate of 1000 µL/min, both particle sizes migrate toward the outer wall with 10 µm particles reaching the outer wall first. Here, we observe that both particles are dragged toward the outer wall due to the absence of resistance in a sheath fluid. The separation efficiency of 10 µm particles was 99% and the efficiency drops to 89% for 15µm particles.


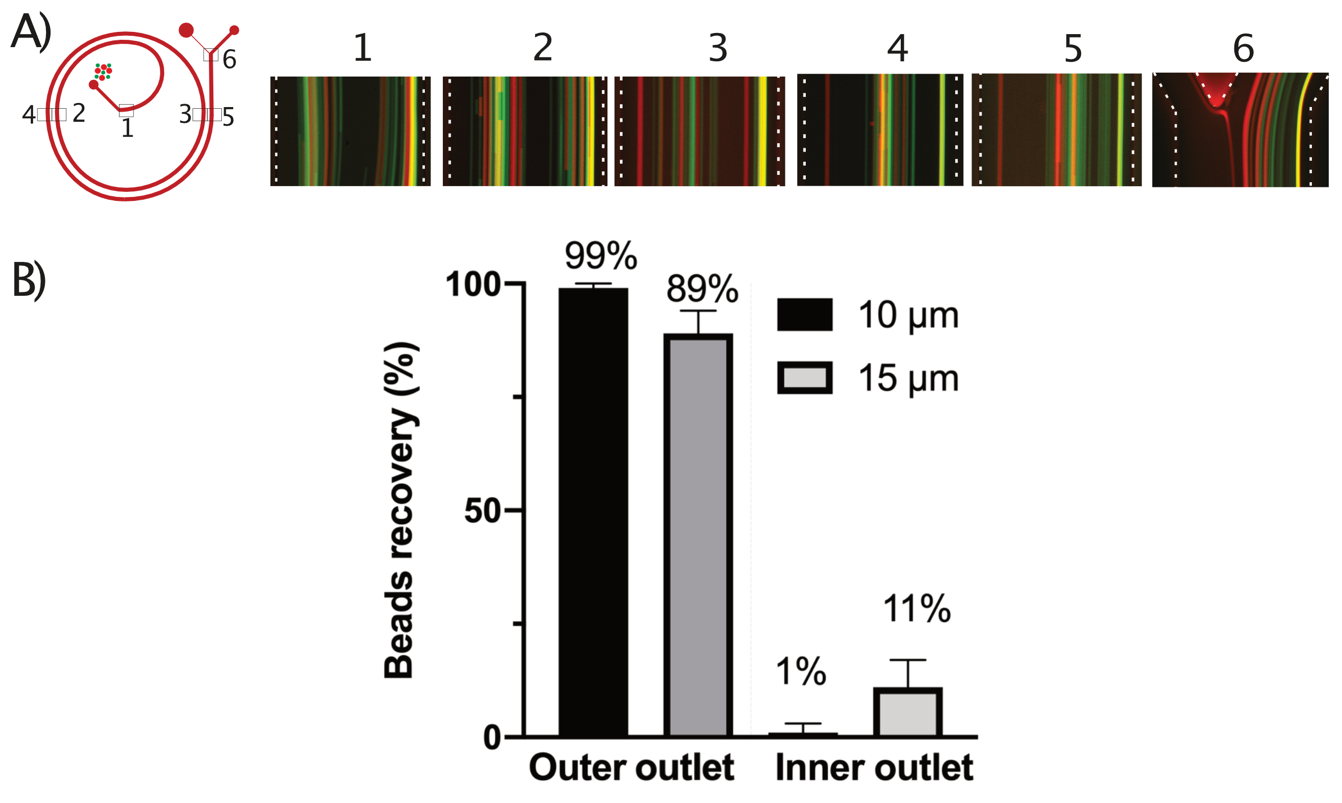


**Figure S4.** The influence of no-sheath fluid on the behavior of 10 (green) and 15 µm (red) particles in a two-turn spiral. a) the influence of lack of sheath flow is shown in 6 different positions along the spiral channel, while in b) the separation efficiency for 10 and 15 um particles is shown.

1. **Comparison between Newtonian and non-Newtonian**

Using the two turn spiral, we evaluated inertial focusing in flow through Newtonian fluid. Using 10 and 15 µm particle sizes, different flow rates and different (with sheath and without sheath) flow conditions were examined (see S5). As expected, the particles could be focused close to the inner when the lift and dean forces are significantly high. As can be seen in Fig. S5, sheath enable better focusing. This is presumably due to the relatively shorter distance particle will travel to find the equilibrium position close to the inner wall. Furthermore, it can be observed that sheath towards the inner wall achieves better separation due to differentially migrating of the smaller particles away from the inner wall for higher flow rates. Hence, by optimizing the flow conditions it should be possible to separate particles in inertial microfluidics.


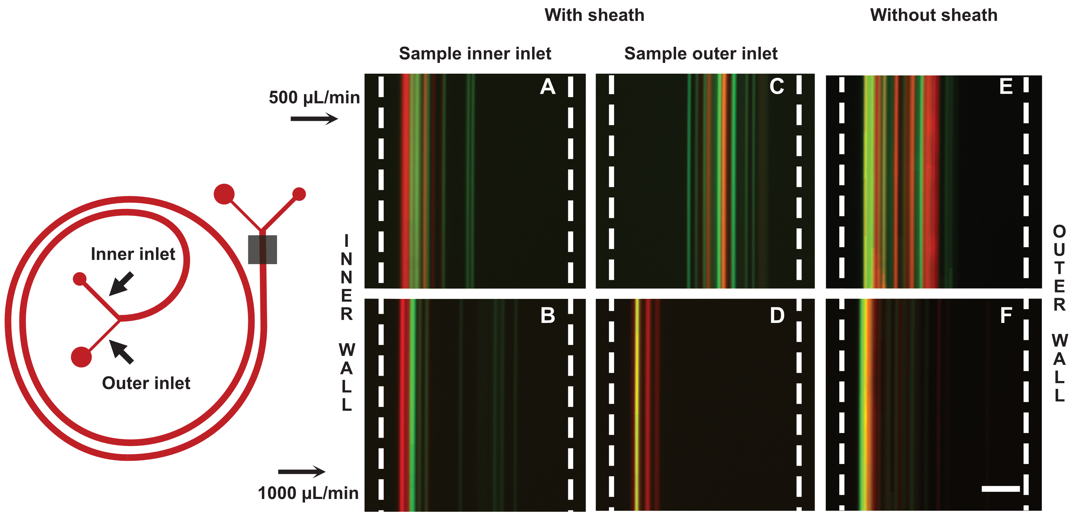


**Figure S5.** Inertial focusing in flow through spiral. Inertial focusing of 10 µm(green) and 15 µm (red) particles in flow through two turn spiral with and without sheath. (A,B) Shows particle sheathing towards inner wall and (C,D) towards outer wall. Upper panel shows flow rate of 500 µL/min (sheath flow rate of 450 µL/min and sample at 50 µL/min) and lower panel shows flow rate of 1000 µL/min (sheath flow rate of 950 µL/min and sample at 50 µL/min). (E,F) No sheath and total flow rate of 500µL/min (upper panel) and 1000 µl/min (lower panel). The flow condition where the particles are sheathed towards the inner wall and high flow rate (B) enables differentially migration of the smaller 10 µm particles for separation. Scale bar: 100µm.
